# A retrospective study of in-hospital mortality in patients with idiopathic pulmonary fibrosis between 2015 and 2018

**DOI:** 10.1097/MD.0000000000023143

**Published:** 2020-11-20

**Authors:** Michael T. Durheim, Jennifer Judy, Shaun Bender, Megan L. Neely, Dorothy Baumer, Scott B. Robinson, Craig S. Conoscenti, Thomas B. Leonard, Howard M. Lazarus, Scott M. Palmer

**Affiliations:** a Duke Clinical Research Institute; b Duke University Medical Center, Durham, North Carolina, USA; c Department of Respiratory Medicine, Oslo University Hospital - Rikshospitalet, Oslo, Norway; d Premier Inc., Charlotte, North Carolina; e Boehringer Ingelheim Pharmaceuticals, Inc., Ridgefield, Connecticut, USA.

**Keywords:** database, diffuse parenchymal lung diseases, hospitalization, interstitial lung diseases

## Abstract

Hospitalizations are common in patients with idiopathic pulmonary fibrosis (IPF) and are associated with high mortality. We used data from the Premier Healthcare Database to determine in-hospital mortality rates and the factors associated with in-hospital mortality in patients with IPF in the era of approved antifibrotic drugs.

The Premier Healthcare Database is a detailed and broadly representative database of hospital admissions and discharges in the US. Patients with IPF who were hospitalized between 1 January 2015 and 28 February 2018 were identified using a diagnostic algorithm comprising International Classification of Diseases -9 and International Classification of Diseases -10 diagnostic codes and billing data. Associations between patient-, hospital- and treatment-related factors and a composite outcome of death during the index visit, lung transplant during the index visit but >1 day after admission, or death during a readmission within 90 days of the index visit were analyzed using logistic regression.

The cohort comprised 9667 hospitalized patients with IPF. In total, 1414 patients (14.6%) met the composite outcome: 1036 (10.7%) died during the index visit, 371 (3.8%) died during a readmission within 90 days; 7 (0.1%) underwent lung transplant >1 day after admission. Factors significantly associated with a higher risk of the composite outcome included mechanical ventilation (odds ratio 6.41 [95% CI: 5.24, 7.84]), admission to the intensive care unit (1.73 [1.49, 2.00]), attendance by a critical care physician (2.12 [1.33, 3.38]), older age (1.20 [1.12, 1.28] per 10-year increase), and use of intravenous steroids (1.16 [1.00, 1.34]), intravenous antibiotics (1.49 [1.22, 1.83]) and opioids (3.41 [2.95, 3.93]). Factors significantly associated with a lower risk of the composite outcome included female sex (0.70 [0.61, 0.80]), comorbid chronic obstructive pulmonary disease (0.69 [0.60, 0.78]), attendance by a family medicine physician (0.67 [0.48, 0.94]) or internal medicine physician (0.59 [0.46, 0.75]), and use of oral steroids (0.62 [0.51, 0.77]), statins (0.76 [0.67, 0.87]) and proton pump inhibitors (0.80 [0.70, 0.92]).

In conclusion, patients with IPF are at risk of mortality during a hospital stay or readmission within 90 days, particularly those who receive mechanical ventilation.

## 1 Introduction

Idiopathic pulmonary fibrosis (IPF) is a chronic fibrosing interstitial lung disease characterized by progressive loss of lung function and high mortality.^[1]^ IPF predominantly affects adults over the age of 60 years and is frequently associated with cardiovascular and respiratory comorbidities.^[2–4]^ Hospitalizations are common in patients with IPF and are associated with high mortality.^[5–9]^

The Premier Healthcare Database (PHD) is a detailed and broadly representative administrative dataset that includes over 20% of hospital admissions and discharges in the US. In an earlier analysis of the PHD, among 6665 patients with IPF who were hospitalized between October 2011 and October 2014, in-hospital mortality was approximately 14% and average length of hospital stay was approximately 5 days.^[9]^ Two antifibrotic drugs, nintedanib and pirfenidone, were approved for the treatment of IPF in the US in October 2014. In this study, we sought to determine in-hospital mortality in the era after the availability of antifibrotic drugs. We used the PHD to estimate the rate of in-hospital mortality, the length of hospital stay and the rate of hospital readmission among patients with IPF who had a discharge date between January 2015 and February 2018. We also determined patient-, hospital- and treatment-related factors associated with in-hospital mortality, longer hospital stay and readmission.

## 2 Methods

### 2.1 Study design

This was an observational, retrospective cohort study of patients with IPF who were hospitalized at 740 hospitals in the US. The study population was selected using a diagnostic algorithm comprising diagnostic and procedure codes and billing data.

### 2.2 Patients

Eligible patients had IPF, were ≥50 years old and had a hospitalization discharge date between 1 January 2015 and 28 February 2018. The diagnosis of IPF was based on a primary or secondary International Classification of Diseases (ICD)-9 diagnosis code of 516.3 or 516.31 (for visits prior to 1 October 2015) or an ICD-10 diagnosis code of J84.111 or J84.112 (for visits on or after 1 October 2015) and a billing code for chest CT and/or lung biopsy ≤3 years prior to the index hospitalization. Exclusion criteria were any ICD-9 or ICD-10 code for discharge diagnoses representing an alternative cause of interstitial lung disease during the index hospitalization and an ICD-9 or ICD-10 procedure code for lung transplant surgery (33.5; Z94.2, 0BY%) that occurred within the first day of hospitalization. Manual review of patient charts was conducted in a subgroup of 209 patients from 10 hospitals (5 large [>300 beds] and 5 small [<300 beds], 8 urban and 2 rural, 9 non-teaching and 1 teaching) in a single healthcare system to calculate the positive predictive value (PPV) of the diagnostic algorithm. The PPV was calculated as the number of cases of IPF confirmed by manual chart review divided by the number of cases identified as IPF based on the code-based inclusion criteria. The final study protocol, in conjunction with other documents, were approved by the central Institutional Review Board (IRB) (Shulman/Advarra) prior to manual chart review. Participating hospitals obtained local IRB approval if required.

### 2.3 Outcomes

The primary outcome was a composite of death during the index visit, lung transplant during the index visit but >1 day after admission, or death during a readmission (to the same hospital) within 90 days of the index visit. Patients undergoing transplant >1 day after admission were included as they were admitted for a reason other than a planned transplant (i.e., transplant surgery >1 day after admission likely reflects an urgent, rather than elective, procedure). Secondary outcomes were length of stay in hospital during the index visit and readmission to hospital within 90 days of the index visit. The first hospital visit during the study period was considered the index visit (ie, each patient was only counted once, irrespective of readmission).

### 2.4 Patient-, hospital- and treatment-related factors

Patient-related factors included age, sex, race and primary payer. Comorbidities were identified based on ICD codes (see Table, Supplemental Content). Hospital-related factors included region (South, Midwest, West, or Northeast), population served (urban or rural), and the specialty of the attending physician (pulmonary medicine, family medicine, internal medicine/hospitalist, critical care/intensivist or other). With regards to diagnostic tests and interventions, mechanical ventilation, chest high-resolution computed tomography, lung biopsy and bronchoscopy were identified based on ICD and Current Procedural Terminology/Healthcare Common Procedure Coding System codes (see Table, Supplemental Content). Admission to the intensive care unit (ICU) was identified based on room and board billing. Treatments of interest included antibiotics (oral or intravenous [IV]), steroids (oral or IV), IV heparin, statins, proton-pump inhibitors, anticoagulants and opiates. Data on the use of these medications were obtained from billing descriptions.

### 2.5 Statistical analyses

Multivariable logistic regression was used to determine associations between patient-, hospital- and treatment-related factors and the primary outcome, and between these factors and readmission within 90 days of the index visit. A generalized linear model with a negative binomial distribution was used to determine associations between patient-, hospital- and treatment-related factors and length of stay during the index visit. Models were built using bidirectional stepwise variable selection. An alpha value of 0.01 was considered statistically significant in the process of variable selection.

## 3 Results

### 3.1 Patients

**Figure 1:**
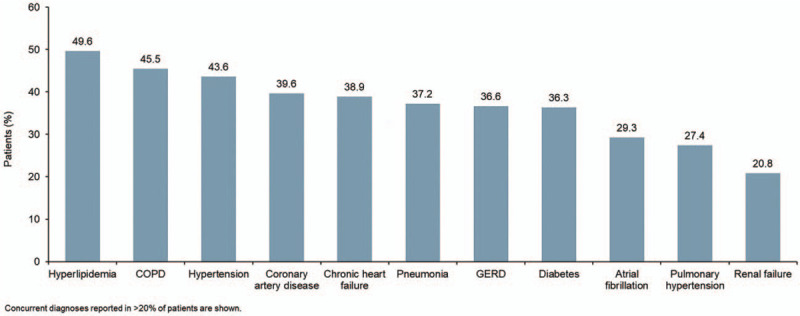
Concurrent diagnoses among hospitalized patients with IPF from January 1, 2015 to February 28, 2018. COPD = chronic obstructive pulmonary disease, GERD = gastroesophageal reflux disease.

**Figure 2:**
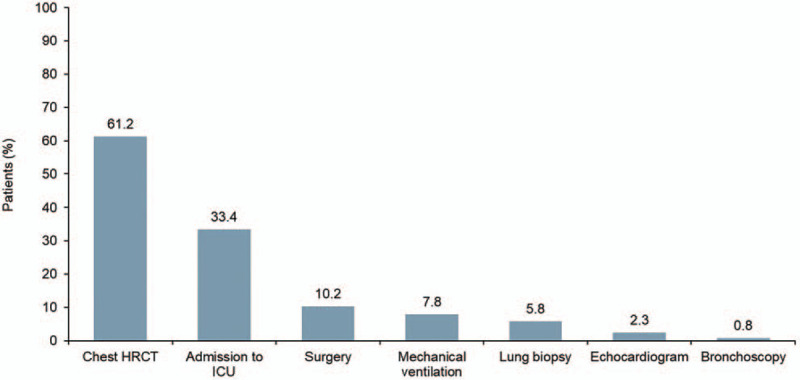
Diagnostic tests and interventions among hospitalized patients with IPF from January 1, 2015 to February 28, 2018. HRCT = high-resolution computed tomography, ICU = intensive care unit.

**Figure 3:**
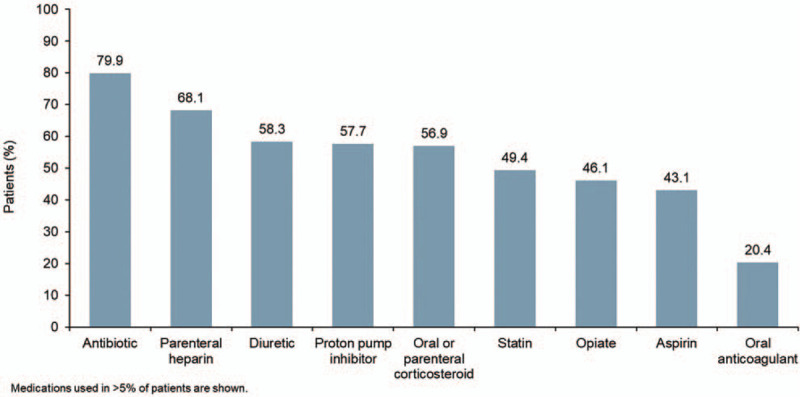
Medication use among hospitalized patients with IPF from January 1, 2015 to February 28, 2018.

The cohort comprised 9667 hospitalized patients with IPF (Table 1). Median age was 76 years, 57.1% of patients were male, and 82.9% of patients were white. The most common concurrent diagnoses were hyperlipidemia and chronic obstructive pulmonary disease (COPD) (Fig. 1). Approximately a third of patients (33.4%) were admitted to the ICU and mechanical ventilation was performed in 7.8% of patients (Fig. 2). The most common medications used were antibiotics (79.9%) and parenteral heparin (68.1%) (Fig. 3).

**Table 1:** Characteristics of hospitalized patients with IPF in the PHD from 1 January, 2015 to February 28, 2018 (n = 9667).

Age, yr, median (Q1, Q3)	76 (68, 83)
Male, n (%)	5523 (57.1)
Race, n (%)	
White	8018 (82.9)
Black/African-American	617 (6.4)
Other	1032 (10.7)
Primary payer, n (%)	
Medicare	7944 (82.2)
Managed care	786 (8.1)
Medicaid	451 (4.7)
Commercial	202 (2.1)
Other	284 (2.9)
Hospital location – US region, n (%)	
South	4469 (46.2)
Midwest	2208 (22.8)
West	1830 (18.9)
Northeast	1160 (12.0)
Attending physician specialty, n (%)	
Internal medicine/hospitalist	6936 (71.7)
Family medicine	670 (6.9)
Pulmonary medicine	521 (5.4)
Critical care/intensivist	150 (1.6)
Other	1390 (14.4)
Teaching hospital, n (%)	3924 (40.6)
Population served by hospital, n (%)	
Urban	8540 (88.3)
Rural	1127 (11.7)

IPF = idiopathic pulmonary fibrosis, PHD = Premier Healthcare Database.

Using a broad definition, where “pulmonary fibrosis” or “idiopathic pulmonary fibrosis [IPF]” was adequate for confirmation and none of the alternative forms of interstitial lung disease was present, 174 patients were confirmed to have IPF, which equated to a PPV of 83.3%. When based on a definition that required use of the term “idiopathic” in the medical record for confirmation of the diagnosis, the PPV was 66.0%.

### 3.2 In-hospital mortality

**Figure 4:**
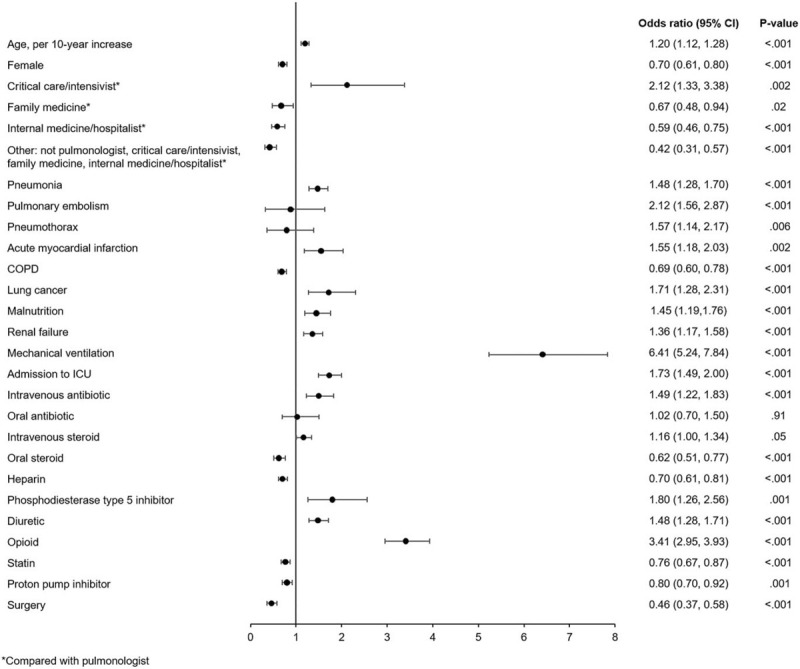
Associations between patient-, hospital- and treatment-related factors and in-hospital mortality or lung transplantation in patients with IPF from January 1, 2015 to February 28, 2018. COPD = chronic obstructive pulmonary disease, ICU = intensive care unit.

A total of 1414 patients (14.6%) met the primary outcome: 1036 patients (10.7%) died during the index visit, 371 patients (3.8%) died during a readmission within 90 days and 7 patients (0.1%) underwent lung transplantation during the index visit but >1 day after admission. Factors significantly associated with a higher risk of in-hospital mortality or lung transplantation included mechanical ventilation (odds ratio (OR) 6.41 [95% CI: 5.24, 7.84]), admission to the ICU (OR 1.73 [95% CI: 1.49, 2.00]), attendance by a critical care physician (OR 2.12 [95% CI: 1.33, 3.38]), older age (OR 1.20 [95% CI: 1.12, 1.28] per 10-year increase), and use of IV steroids (OR 1.16 [95% CI: 1.00, 1.34]), IV antibiotics (OR 1.49 [95% CI: 1.22, 1.83]) and opioids (OR 3.41 [95% CI: 2.95, 3.93]) (Fig. 4).

Factors significantly associated with a lower risk of in-hospital mortality or lung transplantation included female sex (OR 0.70 [95% CI: 0.61, 0.80]), comorbid COPD (OR 0.69 [95% CI: 0.60, 0.78]), attendance by a family medicine physician (OR 0.67 [95% CI: 0.48, 0.94]) or internal medicine physician (OR 0.59 [95% CI: 0.46, 0.75]), and use of oral steroids (OR 0.62 [95% CI: 0.51, 0.77]), statins (OR 0.76 [95% CI: 0.67, 0.87]) and proton pump inhibitors (OR 0.80 [95% CI: 0.70, 0.92]) (Fig. 4).

### 3.3 Length of stay

**Figure 5:**
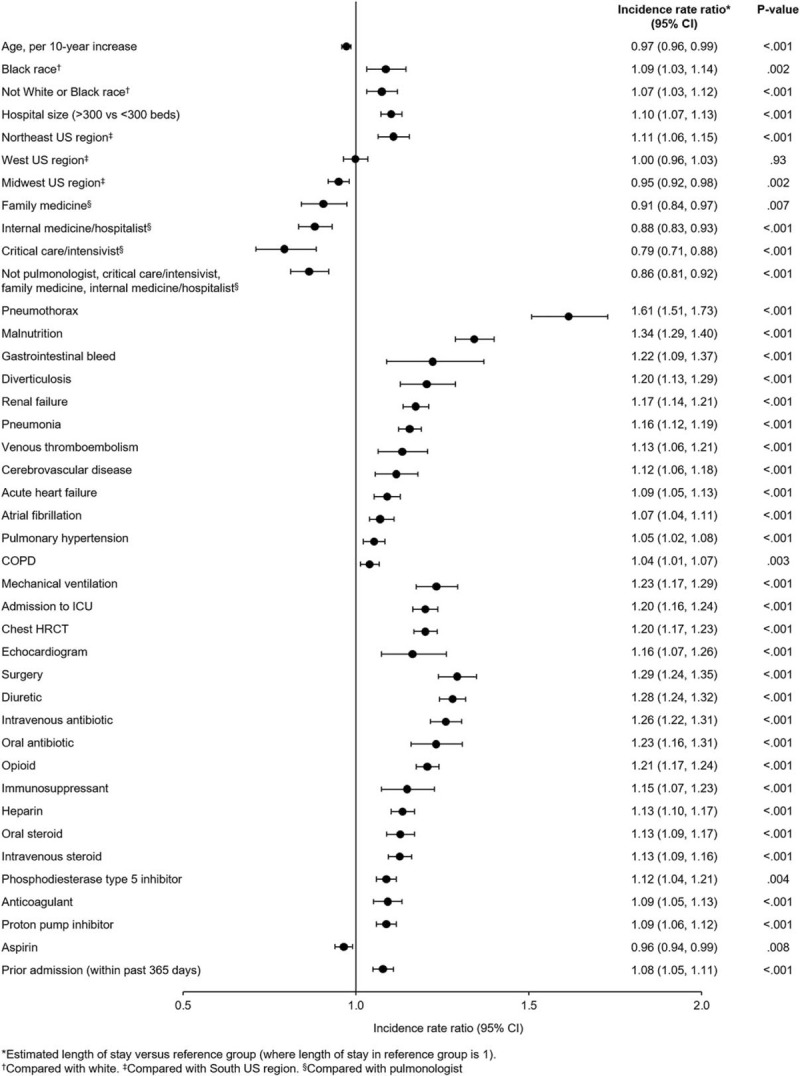
Estimated lengths of stay in hospital among patients with IPF from January 1, 2015 to February 28, 2018 based on patient-, hospital- and treatment-related factors. COPD = chronic obstructive pulmonary disease, HRCT = high-resolution computed tomography, intensive care unit.

Median (Q1, Q3) length of stay in hospital was 5 (3, 8) days: 7 (4, 13) days for patients who died in hospital and 5 (3, 8) days for patients who did not. Median (Q1, Q3) length of stay in the ICU was 4 (2, 7) days: 5 (3, 10) days for patients who died in hospital and 3 (2, 6) days for patients who did not. Patients diagnosed with pneumothorax had an estimated 61% longer stay in hospital than those who were not, while patients diagnosed with pneumonia had an estimated 16% longer stay in hospital than those who were not (Fig. 5). Patients admitted to the ICU had an estimated 20% longer hospital stay, and those who received mechanical ventilation an estimated 23% longer hospital stay, than those who did not (Fig. 5). Patients who had a chest high-resolution computed tomography had an estimated 20% longer hospital stay than those who did not (Fig. 5).

### 3.4 Readmission

**Figure 6:**
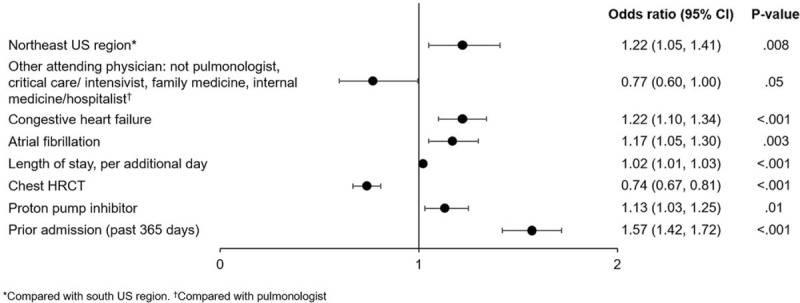
Associations between patient-, hospital- and treatment-related factors and 90-day readmission in patients with IPF from January 1, 2015 to February 28, 2018. HRCT = high-resolution computed tomography.

A total of 2756 patients (28.5%) were readmitted to hospital within 90 days of the index visit; 371 patients (3.8%) died in hospital during that visit. Factors significantly associated with a higher risk of 90-day readmission included admission within 365 days prior to the index visit for any reason (OR 1.57 [95% CI: 1.42, 1.72]), concurrent congestive heart failure (OR 1.22 [95% CI: 1.10, 1.34]), concurrent atrial fibrillation (OR 1.17 [95% CI: 1.05, 1.30]), and use of proton-pump inhibitors (OR 1.13 [95% CI: 1.03, 1.25]) (Fig. 6).

## 4 Discussion

In an analysis of 9667 patients with IPF hospitalized between January 2015 and February 2018, 14.6% died in hospital, underwent lung transplant, or died during a readmission within 90 days. This rate was almost the same as that observed among 6665 patients with IPF in the PHD who were hospitalized between October 2011 and October 2014 (14.4%) that is, in the period prior to the US approval of antifibrotic drugs.^[9]^ Similar rates of in-hospital mortality in patients with IPF have been reported in the Nationwide Inpatient Sample between 2009 and 2011 (14.4%),^[8]^ the Spanish national hospital discharge database between 2004 and 2013 (14.8% in 2004, 13.7% in 2013)^[10]^ and the French hospital discharge database in 2008 (12.8%).^[7]^

Consistent with our earlier analysis of data from the PHD,^[9]^ in this study, mechanical ventilation, admission to ICU, attendance by a critical care physician, and use of IV steroids, IV antibiotics and opioids were associated with a higher risk of in-hospital mortality, likely because these factors reflect care associated with advanced disease. Conversely, female sex, comorbid COPD, and use of oral steroids, statins and proton-pump inhibitors were associated with a lower risk of in-hospital mortality. Data from the Spanish national hospital discharge database in 2014 and 2015 also showed that the presence of COPD was associated with a lower risk of in-hospital mortality in patients with IPF (OR 0.67 [95% CI: 0.56, 0.80]).^[11]^ The reasons for this are not clear, but it might be that patients who have comorbid IPF and COPD and who are hospitalized due to an exacerbation of their COPD have a lower rate of in-hospital mortality, as exacerbations of COPD are not as life-threatening as exacerbations of IPF. Notably, mechanical ventilation was associated with a greater than 6-fold risk of in-hospital mortality. Other retrospective studies have also reported high rates of in-hospital mortality in patients with IPF who were mechanically ventilated, ranging from 55.7% to 74.1%.^[5,12]^ In our 2 analyses of the PHD, 7.8% of the patients hospitalized from 2015 to 2018 were mechanically ventilated, compared with 10.0% of patients who were hospitalized from 2011 to 2014, suggesting that the use of mechanical ventilation in patients with IPF may be declining. Consistent with this finding, data from the Nationwide Inpatient Sample showed a small decline in the use of mechanical ventilation from 2009 to 2011 among 22,350 hospitalized patients with IPF (12.1% to 10.7%).^[12]^

The median length of hospital stay in our study was 5 days. This is consistent with our earlier study based on the PHD,^[9]^ but lower than observed in other observational studies, in which the mean or median length of stay ranged from 8.3 to 10.6 days.^[5,7,10,13]^ In our study, length of stay was longer in patients with pneumothorax or pneumonia, and in patients admitted to the ICU or receiving mechanical ventilation. Approximately 30% of patients hospitalized in our study were readmitted to hospital within 90 days, with admission to hospital within the previous year the strongest predictor of readmission.

Our analysis was based on a large, detailed and diverse database that can be considered broadly representative of patients with IPF across the US. An interesting feature of our analysis is the extension of the previous diagnostic algorithm to identify patients with IPF based on the ICD-10 coding system. We performed additional chart level adjudication and confirmed a high PPV for IPF using this approach (to our knowledge the first time this has been done within the ICD-10 coding system). A limitation of our analysis is that the diagnosis of IPF was based on administrative data. Therefore, patients who had IPF but did not have an ICD-9 or -10 code for IPF would not have been identified. Similarly, ICD-9 or -10 codes were used to identify concurrent diagnoses representing both acute and chronic conditions managed during the hospital admissions, but cannot identify the primary cause of the admission, nor can such codes identify acute exacerbations of IPF, which are likely to affect in-hospital mortality substantially. Some factors known to be associated with mortality in patients with IPF (e.g. poor lung function, use of supplementary oxygen) were not evaluated in our analysis. Readmissions were only based on readmissions to the same hospital, which likely resulted in an underestimation of the readmission rate. Data from placebo-controlled trials have shown that treatment with nintedanib reduces the risk of acute exacerbations of IPF^[14]^ and treatment with pirfenidone reduces the risk of respiratory-related hospitalizations.^[15]^ In addition, a growing body of evidence suggests that these antifibrotic therapies prolong life expectancy.^[4,16–19]^ A limitation of our study was that as the PHD relies on hospital master billing records to identify drug use, and antifibrotic drugs are usually managed and billed through specialty pharmacies, the use of antifibrotic drugs could not be accurately assessed at the patient level. As such, we were unable to determine if clinical outcomes differed among patients who were treated or not treated with antifibrotic drugs.

In conclusion, data from the PHD, a large database representative of hospitalized patients in the US, showed that approximately 15% of patients with IPF who were hospitalized from 1 January 2015 to 28 February 2018 died during a hospital stay or a readmission within 90 days. The risk of in-hospital mortality was higher in patients who were admitted to the ICU, attended by a critical care physician, received mechanical ventilation, or were being treated with IV steroids, IV antibiotics and opioids.

## Author contributions

**Data curation:** Jennifer Judy, Dorothy Baumer, Scott B Robinson.

**Formal analysis:** Shaun Bender, Megan L Neely.

**Methodology:** Michael T Durheim, Jennifer Judy, Shaun Bender, Megan L Neely, Dorothy Baumer, Scott B Robinson, Craig S Conoscenti, Thomas B Leonard, Scott M Palmer.

**Project administration:** Jennifer Judy, Dorothy Baumer, Scott B Robinson.

**Writing – original draft:** Michael T Durheim.

**Writing – review and editing:** Jennifer Judy, Shaun Bender, Megan L Neely, Dorothy Baumer, Scott B Robinson, Craig S Conoscenti, Thomas B Leonard, Howard M Lazarus, Scott M Palmer.

## Supplementary Material

Supplemental Digital Content
