# Supplementary material for: A retrospective study of in-hospital mortality in patients with idiopathic pulmonary fibrosis between 2015 and 2018
Source: Medicine (Baltimore). 2020 Nov 20;99(47):e23143. doi: 10.1097/MD.0000000000023143 (PMC7676591; doi:10.1097/MD.0000000000023143)
Supplement: Supplemental Digital Content [file medi-99-e23143-s001.docx]

**Supplemental content for Durheim MT et al. In-hospital mortality in patients with idiopathic pulmonary fibrosis between 2015 and 2018**

**Table**

| **Characteristic** | **Identification codes/source data** | | |
| --- | --- | --- | --- |
|  | **ICD-9 codes** | **ICD-10 codes** | **CPT/HCPCS Codes** |
| Mechanical ventilation |  |  |  |
| Invasive | 96.7, 96.70, 96.71, 96.72, 31.2, 31.21, 31.29, 96.04, 96.05, V44.0 | 5A1935Z, 5A1945Z, 5A1955Z, Z93.0, Z43.0, 0BH17EZ, 0B717DZ, 0B718DZ, 0BH07DZ, 0BH07YZ, 0BH172Z, 0BH17YZ, 0BH182Z, 0BH18YZ, 0BHK7YZ, 0BHK8YZ, 0BHL7YZ, 0BHL8YZ, 0WHQ7YZ | K0165, 31502, 31600, 31601, 31603, 31605, 31610, 31611, 31612, 31613, 31614 |
| Unknown | V46.1, V46.11, V46.12, V46.13, V46.14, 997.31 | Z99.11, Z99.12, J95.850, J95.851 |  |
| Chest CT; HRCT |  |  | 71260, 71250, 71270 |
| Lung biopsy | 33.25–33.28, 33.27 | 0BBC0ZX, 0BBC3ZX, 0BBC4ZX, 0BBC7ZX, 0BBC8ZX, 0BBD0ZX, 0BBD3ZX, 0BBD4ZX, 0BBD7ZX, 0BBD8ZX, 0BBF0ZX, 0BBF3ZX, 0BBF4ZX, 0BBF7ZX, 0BBF8ZX, 0BBG0ZX, 0BBG3ZX, 0BBG4ZX, 0BBG7ZX, 0BBG8ZX, 0BBH0ZX, 0BBH3ZX, 0BBH4ZX, 0BBH7ZX, 0BBH8ZX, 0BBJ0ZX, 0BBJ3ZX, 0BBJ4ZX, 0BBJ7ZX, 0BBJ8ZX, 0BBK0ZX, 0BBK3ZX, 0BBK4ZX, 0BBK7ZX, 0BBK8ZX, 0BBL0ZX, 0BBL3ZX, 0BBL4ZX, 0BBL7ZX, 0BBL8ZX, 0BBM0ZX, 0BBM3ZX, 0BBM4ZX, 0BBM7ZX, 0BBM8ZX, 0BBN0ZX, 0BBN3ZX, 0BBN4ZX, 0BBN8ZX, 0BBP0ZX, 0BBP3ZX, 0BBP4ZX, 0BBP8ZX | 32098, 32095, 32405, 32096, 32607, 32602 |
| Bronchoscopy |  |  | 31630, 31625, 31628, 31632, 31660, 31661, 31622, 31623, 31624, 31652, 31653, 31654, 31635, 31629, 31633, 31645, 31631, 31636, 31637, 31638 |
| Echocardiogram | 37.28, 11.24, 88.73 | B24% |  |
| Comorbidities |  |  |  |
| Acute heart failure | 428.21, 428.23, 428.31, 428.33, 428.41, 428.43 | I50.21, I50.23, I50.31, I50.33, I50.41, I50.43 |  |
| Atrial fibrillation | 427.3xx | I48% |  |
| Cerebrovascular disease | 430.xx – 438.xx | I60%, I61%, I62%, I63%, I65%, I66%, I67%, I68%, I69%, G45% |  |
| Chronic obstructive pulmonary disease | 490.xx – 491.xx, 492.xx, 494.xx, 496.xx | J43%, J44% |  |
| Chronic heart failure | 428.xx | 'I50.1', 'I50.20', 'I50.21', 'I50.22', 'I50.23', 'I50.30', 'I50.31', 'I50.32', 'I50.33', 'I50.40', 'I50.41', 'I50.42', 'I50.43', 'I50.9' |  |
| Coronary artery disease | 414.01, 414.0x | I25% |  |
| Diabetes | 250.xx | E08% – E13% |  |
| Diverticulosis | 562.xx | K57% |  |
| Gastroesophageal reflux disease | 530.81x | K21% |  |
| Gastrointestinal bleed | 578.9 | K92.2 |  |
| Hyperlipidaemia | 272.4x, 272.2xx | E78.4, E78.5, E78.2 |  |
| Hypertension | 401.xx | I10 |  |
| Lung cancer | 162.xx | C33, C34% |  |
| Malnutrition | 262, 263, 263.0, 263.1, 263.8, 263.9x | E43, E44%, E46 |  |
| Pneumonia | 481, 482.0, 482.1, 482.2, 482.30, 482.31, 482.32, 482.39, 482.40, 482.41, 482.42, 482.49, 482.81, 482.82, 482.83, 482.84, 482.89, 482.9, 483.1, 483.8, 484.8, 485, 486, 487.0, 487.1, 487.8, 488.0, 488.1 | J13, J18.1, J18, J18.2, J18.8, J15.0, J15.1, J14, J15.4, J15.4, J15.3, J15.4, J15.20, J15.211, J15.212, J15.29, J15.8, J15.5, J15.6, A48.1, J15.8, J15.9, J16.0, J16.8, J17, J18.0, J18.9, J11.00, J12.9, J10.1, J11.1, J11.2, J11.81, J11.89, J09.X1, J09.X2, J09.X3, J09.X9, J10.08, J10.1, J09.X3, J09.X9, J10.0, J10.00, J10.01, J10.2, J10.8, J10.81, J10.82, J10.83, J10.89, J11, J11.0, J11.08, J11.8, J11.82, J11.83, J12, J12.0, J12.1, J12.2, J12.3, J12.8, J12.81, J12.89 |  |
| Pneumothorax | 512.xx | J93% |  |
| Renal failure | 584.xx | N17% |  |
| Venous thromboembolism | 415.1, 453.40 | I82%, I26% |  |

CPT, Current Procedural Terminology; HCPCS, Healthcare Common Procedure Coding System; ICD, International Classification of Diseases.
